# An AI for diseases or a sick AI? The epistemological pathology of big data overload and the loss of statistical significance

**DOI:** 10.3389/frai.2026.1961525

**Published:** 2026-09-09

**Authors:** Marco Roccetti

**Affiliations:** Department of Computer Science and Engineering Bologna, University of Bologna, Bologna, Italy

**Keywords:** big medical data, Garwood’s antidote, Poisson distribution, rare events, sick artificial intelligence, statistical significance

## Abstract

Modern clinical epidemiology and artificial intelligence are increasingly driven by an idealized premise: the belief that massive databases and advanced machine learning can redeem causal inference from observational uncertainty. Behind the facade of multi-million-record cohorts, however, a profound epistemological crisis festers, revealing a potentially sick artificial intelligence. Modern analytical systems leverage massive sample sizes to generate ultra-significant asymptotic *p*-values that reflect mathematical outcomes rather than biological reality. This article diagnoses this systemic pathology, the uncritical application of Gaussian asymptotic statistics to sparse, discrete, and rare medical event counts governed by Poisson distributions. To cure this condition, we introduce a methodological antidote: an inference stress test that repurposes the exact lower boundary of the exact Poisson confidence interval as a formal null benchmark, utilizing an event-anchored standard error. Across three clinical case studies, spanning autoimmune dermatology baselines, expanded matching cohorts, and oncological lifestyle investigations, our stress test provides evidence that nominal asymptotic significance may sometimes fail to clear the structural resistance threshold, revealing the underlying fragility of scale-induced signals. This proposed framework serves as an essential epistemological filter, separating genuine biological discovery from digital outcomes and ensuring that future medical AI systems learn only from structurally validated knowledge.

## 1 Introduction

In recent years, the methodological debate in medicine has been overwhelmed by a promise: the illusion that combining large-scale databases, machine learning algorithms, and advanced balancing techniques (such as *Propensity Score Matching*) can redeem causal inference from observational uncertainty. Yet, behind the facade of massive cohorts exceeding millions of individuals, a profound crisis festers, a true *epistemological crisis* afflicting both modern artificial intelligence systems and clinical epidemiology, drowning in a mass of useless, unvetted data and suffering from a progressive loss of true significance (Habehh and Gohel, 2021; Alhumaidi et al., 2025; Roccetti, 2026a).

The true patient is now not only the individual at the center of our attention, but the analytical instrument itself: a system afflicted by structural blindness toward biological reality, reduced to operating as a purely associative pattern-matcher. This distortion unfolds across two fundamental junctures which can be briefly described as follows.

To begin, in the debate surrounding AI and big clinical data, the first fundamental issue is that models (and massive epidemiological studies built on millions of records) function as uncritical data-matchers: they manipulate vast amounts of information, recombining them into formally coherent and seemingly significant patterns that are nonetheless often detached from biological and causal reality. As a possible consequence, faced with an enormous database (such as a metropolitan cohort of over 3 million individuals), traditional statistical machinery churns out an ultra-significant *p*-value or a PSM-calibrated Hazard Ratio by virtue of the massive computational scale of the dataset. Yet that number often describes a digital instability generated by questionable correlations, not real biological phenomena. It is a potential logical disconnect in clinical inference: formal associations masquerading as causality.

Second, large-number-based medicine has widely adopted an unverified premise: *the larger the sample, the truer the result*. This dogma drives an unconditional trust in tools like Wald’s test or standardized Cox proportional hazards models used to leverage covariates and other subtleties, which inherently presuppose that variability follows an ideal, continuous asymptotic distribution, that is the classic Gaussian curve (Therneau, 1997). However, the problem is that when confronted with millions of records, each medical inquiry concerns *rare events* (which occur statistically infrequently with respect to the enormity of non-positive cases). As a result, the reality of the counts is far from Gaussian: it is sparse, discrete, and governed by the pure stochasticity of a Poisson distribution. Unfortunately, relying on standard asymptotic formulas in this sparse regime risks overlooking the discrete nature of the phenomenon and compressing the underlying fragility of supposed medical signals.

Hence, modern medical engineering and informatics needs to find an epistemological antidote to diagnose and cure this pathology. To achieve that, we must first recognize its symptoms: they are hidden in plain sight within the confidence intervals assigned to associations, those numerical intervals upon which modern AI models are expected to train. Yet, the problem stemming from the issues exposed above is that these confidence intervals are systematically distorted by the inappropriate application of Gaussian asymptotic statistics to discrete, rare phenomena.

Therefore, in this paper, we show through three striking, highly expressive, and conceptually simple examples how to remedy this flaw and construct a structural diagnostic framework, utilizing exact Poisson uncertainty bounds and standardized distance metrics, on which a sane, sure, reliable, clinical AI may be instructed. This approach will allow us to definitively determine whether those data and associations are worthy nourishment for an AI or merely digital instability. It should not be forgotten, in fact, that foundational epidemiological associations and relative findings constitute the core empirical knowledge upon which future medical AI systems will learn, making their structural validation an essential prerequisite to prevent algorithms from ingesting and amplifying scale-induced artifacts.

Before diving into the full analysis, we can outline the core methodology and preview our three case studies with which we will test our method. As to the proposed methodology (reported in Section 2), we will show how resorting to a classical frequentist approach for the exact calculation of the confidence intervals (CI) will set the basis for the problem solution. It is a revisitation of the well known Garwood method, reinterpreted though the introduction of a stress test (i.e., H0 = the lower limit of the Garwood CI calculation) employing a Poisson standard error that applies the same principles of radical accountability and transparency demanded of advanced algorithmic systems (Garwood, 1936).

As to the case studies, we will first prove our approach first on a fascinating, evolving clinical exemplar spanning two distinct large-scale datasets: an initial foundational cohort and its subsequent, expanded multi-million-record iteration, both of which have revealed structural statistical instability beneath their statistical exterior (Kim et al., 2025; Roccetti, 2026b; Kim et al., 2026). Second, we will stress test a critical investigation from the field of oncology, focusing on modifiable lifestyle exposures and outcomes, testing whether apparent risk signals possess full physical consistency or attenuate when evaluated against exact Poisson uncertainty floors and standardized structural benchmarks (Sala et al., 2026).

For all these cases, the stress test we propose takes the polished result extracted from the multi-million-record database, places it under intense scrutiny against the exact lower limit of the true event distribution, and mathematically measures its true inferential resilience. Moving from critical reflections to these calculations means making the leap from theoretical denunciation to precise correction, forcing modern epidemiology and machine learning to finally reckon with the structural limits of their own arithmetic.

## 2 Data and methods

To empirically evaluate the structural fragility of asymptotic estimators across distinct clinical contexts, this Section presents the foundational data inputs and methodological framework for our three case studies. These span autoimmune dermatology, its subsequent large-scale multi-million replication, and modern oncological research concerning lifestyle exposures (e.g., smoking).

### 2.1 Data sources and baseline characteristics

Rather than relying on unvetted asymptotic assumptions, our framework aggregates and standardizes the exact event counts, population sizes, and person-years across three exemplar representative scenarios:

Case Study 1 (Autoimmune Dermatology - Initial Cohort): The initial observational baseline evaluating short-term Vitiligo incidence following COVID-19 vaccination versus a non-vaccinated control group, as reported in (Kim et al., 2025).

Case Study 2 (Autoimmune Dermatology - Extended Cohort): A subsequent 1:4 Propensity Score Matching (PSM) extended analysis utilizing the entire adult population of a major Asian Pacific city across an extended multi-million-record database evaluated at a 1-year follow-up (Kim et al., 2026).

Case Study 3 (Oncology and Moderate Smoking Cohort): A prospective cohort evaluation assessing early-onset cancer risk among light or moderate smokers (current smokers of fewer than 16 cigarettes/day), extracting raw absolute counts and person-years from large-scale European biobank infrastructures (Sala et al., 2026).

The unified baseline parameters for all these three distinct empirical investigations are summarized in Table 1 below.

**Table 1:** Baseline data inputs and exact parameters across the three analytical case studies.

Case study/cohort	Subgroup/arm	Population/person-years (*N* or *PY*)	Observed events (*n* or *Y*)	Observed rate: *p*(*Inc*) or λ)
Vitiligo (initial)	Unvaccinated controls	343,311 (*N*)	23	0.670 (per 10,000)
	Vaccinated cohort	2,086,709 (*N*)	463	2.219 (per 10,000)
Vitiligo (extended)	Unvaccinated controls	2,415,364 (*N*)	314	1.30 (per 10,000)
	Vaccinated cohort	603,841 (*N*)	87	1.44 (per 10,000)
Oncology (smoking)	Light/Moderate smokers (<16 cig/day)*	276,461 (*PY*)	206	0.000745 (rate)

*In the table and subsequent formulations, p(Inc) represents the crude incidence rate expressed per 10,000 individuals for cohort-based event counts (*n*/*N* per 10,000), while λ represents the crude incidence rate expressed per person-year for time-at-risk count data (Y/PY). Parameters for Case study 3 reflect the male cohort subset extracted for the strict stress-test evaluation of light/moderate smoking strata under age 50 of (Sala et al., 2026).

### 2.2 Statistical analysis

In this section, we delineate the statistical methods and corresponding formulas employed to verify the inferential resilience of the study data across all three analytical case studies.

First, we begin by assuming to work with a Normal distribution and thus employing the Wald approximation (like in the original studies (Kim et al., 2025; Kim et al., 2026; Sala et al., 2026)). Hence, we reiterate the standard calculations that inherently assume an asymptotic Gaussian distribution for the test statistic (questionable for the analysis of rare event counts). Under this framework, the Standard Error (*SE*) and the resulting confidence intervals (CI) for an incidence rate *p*(*Inc*) (defined as the number of events per 10,000 subjects, or using rate λ per person-year) can be calculated as per the Equations 1, 2 below (Rothman et al., 2008):


$$\mathrm{SE}=\sqrt{\frac{p(\mathrm{Inc})(1-p(\mathrm{Inc})/10,000)}{N}}\times 10,000$$ (1)

CI=p(Inc)±(1.96×SE) (2)

To assess the inferential resilience of these estimates, we calculate the *Z*-score (using the formula: *p*(Inc)/*SE*)) and the corresponding *p*-value for each cohort, against the null hypothesis H0: *p*(Inc) = 0. This test demonstrates the statistical stability and precision of the observed incidence rates under standard Gaussian assumptions.

However, the use of the Wald Gaussian approximation, while mathematically convenient, tends to artificially refine the inferential output in large datasets. By assuming a Normal distribution, the resulting confidence intervals are usually narrower and the corresponding *p*-values are much lower, which might lead to a perception of heightened accuracy. While this approach reinforces apparent statistical significance, it essentially relies on the tightness of the symmetric distribution, potentially overstating the precision of the point estimate.

To determine whether these results are simply an outcome of model assumptions or a reflection of an underlying reality, we must move beyond asymptotic approximations. To this aim, we shall now evaluate the data using the exact Poisson-based Garwood method; this approach reveals whether the inferential strength of the findings persists when the symmetry constraint is removed, providing a more critical assessment of the observed risk across our three distinct case studies (Garwood, 1936; Agresti and Coull, 1998).

Unlike the Wald standard error, which relies on a symmetric normal approximation of proportions, the natural variability of rare event counts or incidence rates governed by a Poisson process requires a variance structure proportional to the mean. For any observed event count *n* (or *Y*) over a population size *N* (or person-years *PY*), the underlying Poisson standard error used in our stress tests is defined as *SE*(*Poisson*) = $\sqrt{n}$ /*N* × 10,000 (for cohort rates expressed per 10,000 subjects) or, when dealing with person-time data: *SE*(*Poisson*) = $\sqrt{Y}$ /*PY* (for person-year incidence rates λ ). These formulations ensure that the measure of dispersion scales correctly with the absolute sparsity of the events, rather than being artificially deflated by massive non-event denominators.

Moreover, to rigorously assess whether an observed clinical signal possesses genuine physical consistency or is merely an outcome of asymptotic modeling, we need to build a stress test on top of a null benchmark, intended as the lower limit of the exact Poisson distribution (indicated as H0 = *LL*(*Garwood*)). To this aim, the relative *Z*-score comes therefore computed as: *Z* = (*p*(*Inc*) - *LL*(*Garwood*))/*SE*(*Poisson*) (for cohort-based rates), or, equivalently for person-time analyses: *Z* = ( λ - *LL*(*Garwood*))/*SE*(*Poisson*).

By substituting the traditional regression parameters with this exact Poisson stress test, we can measure the true distance between the observed incidence and its absolute worst-case uncertainty floor. If the resulting *Z*-score fails to cross the critical threshold of 1.96 (corresponding to a one-tailed α = 0.05), the initial signal attenuates, revealing the structural fragility of the underlying data.

To introduce, now, the basic principles of the Garwood method, it is worth reminding that for event counts, the distribution is inherently discrete and right-skewed. While large datasets may suggest a Gaussian profile, the exact Confidence Interval (CI) should be derived from the Poisson distribution, which governs the occurrence of discrete events. In this analysis, we employ the Garwood method to map these counts onto a $\chi^{2}$ distribution, ensuring the calculation of precise, non-symmetric boundaries that do not rely on asymptotic assumptions.

Consequently, the lower and upper limits (*LL* and *LU* of Equations 3, 4) of a count CI are defined using the relationship between Poisson parameters and the $\chi^{2}$ distribution with corresponding degrees of freedom (df):


$$\mathrm{LL}=\frac{\chi^{2}(\alpha /2,2n)}{2}$$ (3)

$$\mathrm{LU}=\frac{\chi^{2}(1-\alpha /2,2n+1)}{2}$$ (4)

Where *n* represents the observed event count (or *Y* for person-year data). By using 2*n* and 2(*n* + 1) as degrees of freedom, we account for the Poisson distribution’s lower bound at zero, providing a conservative and mathematically consistent estimation. Essentially, to build these new confidence intervals we proceed as follows.

The lower limit (*LL*) uses $\frac{\chi^{2}(\alpha /2,2n)}{2}$ , with α /2 = 0.025 (capturing the lower 2.5% of possible outcomes of the left tail) and 2*n* degrees of freedom (scaling the count to match the exact $\chi^{2}$ quantiles for the Poisson parameter). The upper limit (*LU*), instead, uses $\frac{\chi^{2}(1-\alpha /2,2n+1)}{2}$ , capturing the upper 2.5% of the right tail and utilizing 2(*n* + 1) degrees of freedom. This exact upper-tail term accounts for the discrete nature of the distribution, providing a conservative ceiling.

In summary, the transformation from *n* to 2*n* or 2(*n* + 1) is the critical step that differentiates an exact Poisson method from a Normal approximation. While the Wald method uses a flat, symmetric standard error, the Garwood method uses these $\chi^{2}$ quantiles to stretch the interval dynamically based on the actual count *n*, correctly accounting for the inherent skewness of rare events.

Now, to obtain the final incidence rates, these exact bounds are normalized by the respective population sizes or person-years for each of our three case studies, as synthesized in Table 2 below.

**Table 2:** Garwood method application and exact parameters across the three case studies.

Case study/cohort	Group/arm	Event (n/Y)	dflow (2n)	dfup 2(n + 1)	*χ* ^0.0252^	*χ* ^0.9752^	LL	LU
Vitiligo Initial	Unvax	23	46	48	29.160	69.023	14.580	34.512
	Vax	463	926	928	841.691	1013.84	420.846	506.920
Vitiligo extended	Unvax	314	628	630	575.240	683.475	287.620	341.738
	Vax	87	174	176	145.360	207.540	72.680	103.770
Oncology	Mod. Smoke	206	412	414	360.480	467.520	180.240	233.760

It is worth noticing now that while for high-frequency counts the Garwood intervals converge toward symmetric Wald intervals, the use of this exact method serves a critical purpose: it validates the inferential resilience of the findings without relying on Gaussian approximations. This rigorous approach confirms whether observed discrepancies in risk are genuine features of the data or merely artifacts of asymptotic statistical frameworks.

At this point, we deem also useful briefly clarifying the nature of this new index, its algebraic derivation, and asymptotic behavior. We reiterate that this procedure does not propose a new formal frequentist hypothesis test in the strict sense. In fact, the exact Garwood lower limit is derived from the observed data *n*, and treating it as a fixed, independently specified null value in a traditional frequentist framework would raise formal challenges regarding distributional assumptions and Type I error calibration.

Instead, our proposed metric is utilized here strictly as a standardized descriptive index of structural distance, a diagnostic load-test measuring how many Poisson standard errors separate the observed estimate from its own exact lower uncertainty boundary.

To substantiate this diagnostic nature of this index and demonstrate its theoretical properties, we can summarize its algebraic structure. To this aim, let $\hat{\lambda }$ = *n*/*T* be the observed rate, *SE* = $\sqrt{n}$ /*T* the Poisson standard error, and *LL*(*Garwood*) = $\frac{\chi^{2}(2n,\frac{\alpha }{\;2})}{2T}$ the exact lower confidence limit, where *T* represents the administrative denominator (population or person-time at risk). Constructing the standardized distance index, yields with Equation 5:


$$Z=\frac{\hat{\lambda }-\mathrm{LL}(\text{Garwood})}{\mathrm{SE}}=\frac{\frac{n}{T}-\frac{\chi^{2}(2n,\frac{\alpha }{2})}{2T}}{\frac{\sqrt{n}}{T}}$$ (5)

As evident from this formulation, the administrative denominator *T* cancels out entirely. The index reduces to a purely discrete, scale-invariant function of the observed event count *n* and its exact $\chi^{2}$ quantile, as shown in Equation 6 below:


$$Z=\frac{n-\frac{1}{2}\;\chi^{2}(2n,\;\frac{\alpha }{2})}{\sqrt{n}}$$ (6)

Because of this exact algebraic cancellation, this index is entirely invariant to denominator inflation caused by massive database scaling. Furthermore, regarding its theoretical distributional behavior, applying standard asymptotic expansions for the $\chi^{2}$ quantile reveals that as *n* increases, *Z* analytically converges toward the standard normal reference threshold (e.g., 1.96, for α = 0.05). Thus, rather than depending on an external frequentist null distribution, the standard normal reference value emerges naturally as the asymptotic limit of the discrete Poisson process itself, providing a theoretical foundation for its interpretation as an invariant structural benchmark. We reiterate, nonetheless, that this metric serves not as a replacement for standard statistical inference, but as an internal diagnostic tool designed to assess finite-count structural resilience independently of administrative scale.

Ultimately, a technical consideration on our distributional choices is warranted. While it is undisputed that binary event counts in fixed cohorts are natively governed by a Binomial distribution, in the rare-event regime where event probabilities are vanishingly small Binomial and Poisson frameworks practically converge. Our exact bounds operate under a pure Poisson assumption as a primary baseline safeguard. Nevertheless, researchers must remain mindful that unmeasured overdispersion, clustering, or dependence can introduce extra-Poisson variability. While our exact Poisson stress test provides a necessary anchor against inflated false positives in massive datasets, accounting for potential data clustering remains an important boundary condition in real-world clinical applications.

## 3 Results

By applying the inference stress test framework we have described before [i.e., H0 = *LL*(*Garwood*)] with a Poisson standard error) across our three distinct clinical scenarios, we have evaluated whether the initial risk signals of the three cases of interest (Kim et al., 2025; Kim et al., 2026; Sala et al., 2026) possess physical consistency or represent digital outcomes generated by massive denominators.

Before showing the obtained results, the following should be reiterated: to ensure analytical consistency, all stress-test evaluations are conducted as one-tailed directional assessments measuring the structural distance between the observed rate and its exact lower uncertainty floor. Furthermore, we adopted the asymptotic threshold of 1.96 (corresponding to the standard 95% confidence level limit) as our primary structural resistance benchmark. The reported *p*-values are hence provided only as indicative, heuristic descriptors to facilitate intuitive interpretation against conventional benchmarks, while our primary focus remains on evaluating whether the structural *Z*-score successfully clears this invariant uncertainty boundary.

### 3.1 Vitiligo – initial cohort

When raw observational counts from the preliminary baseline cohort are subjected to exact Poisson boundaries rather than asymptotic assumptions, the massive initial signals (which yielded Wald *p*-values < 0.0001) face immediate scrutiny regarding their small-cell dispersion and foundational stability which can be calculated as follows.

*Unvaccinated Controls* (*n* = 23, *N* = 343,311): Observed incidence 0.670 per 10,000. The Garwood lower limit yields *LL*(*Garwood*) = 0.4247 per 10,000. With *SE*(*Poisson*) = 0.1397, the stress test Z-score is 1.756 (indicative one-tailed *p* ≅ 0.0395).

*Vaccinated Cohort* (*n* = 463, *N* = 2,086,709): Observed incidence 2.219 per 10,000. The Garwood lower limit yields *LL*(*Garwood*) = 2.0168 per 10,000. With *SE*(*Poisson*) = 0.1031, the stress test Z-score is 1.961 (indicative one-tailed *p* ≅ 0.025).

In summary for this first case, even when initial Wald statistics declare overwhelming significance (*p*-value < 0.0001), exact Poisson bounding shows that the structural margin of resistance hovers close to the 1.96 limit.

### 3.2 Vitiligo – extended multi-million cohort

We now examine the data from the expanded study utilizing 1:4 Propensity Score Matching, advanced covariate balancing, the entire adult population of a major Asian Pacific city, and a 1-year Cox proportional hazards model.

*Unvaccinated Controls* (*N* = 2,415,364): 12-month cumulative incidence *I* = 1.30 per 10,000; *n* ≅ 314 events):

*LL*(*Garwood*) (per 10,000) = (287.62/2,415,364) per 10,000 = 1.191 per 10,000.

*SE*(*Poisson*) = $\sqrt{314}$ /2,415,364 per 10,000 ≅ 0.0734.

Z-score: *Z* = (1.30–1.191) /0.0734 ≅ 1.485 (indicative one-tailed *p* ≅ 0.0688).

*Vaccinated Cohort* (*N* = 603,841): 12-month cumulative incidence *I* = 1.44 per 10,000; *n* ≅ 87 events).

*LL*(*Garwood*) (per 10,000) = (72.68/603,841) per 10,000 ≅ 1.204 per 10,000.

*SE*(*Poisson*) = ( $\sqrt{87}$ /603,84) per 10,000 ≅ 0.1545.

Z-score: *Z* = ((1.44–1.204)/0.1545) ≅ 1.527 (indicative one-tailed *p* ≅ 0.0634).

These results for the extended cohorts are also summarized in Table 3.

**Table 3:** Stress test summary (extended cohort – vitiligo at 12 months).

Parameter	Controls (*N* = 2.4 M)	Vaccinated (*N* = 603 k)
Estimated events (*n*)	314	87
Observed incidence (per 10 k)	1.30	1.44
*H0* = *LL* (*Garwood*)	1.191	1.204
*SE* (*Poisson*)	0.0734	0.1545
Stress test Z-score	1.485	1.527
Indicative *p*	0.0688	0.0634

In conclusion, for this second Vitiligo case, even after deploying 1:4 PSM, massive covariate balancing, and a 1-year Cox model boasting asymptotic *p*-values below 0.0001 (Kim et al., 2025; Kim et al., 2026), the stress test exposes the structural fragility of the dataset: both *Z*-scores do not clear the asymptotic 1.96 resistance threshold, providing some evidence that the risk signals hover below the Poisson uncertainty floor.

### 3.3 Oncology and moderate smoking cohort

To further test the limits of asymptotic survival models in a different low-incidence setting, we examine early-onset cancer risks among light or moderate smokers (current smokers of fewer than 16 cigarettes/day), utilizing the male cohort data exposed in (Sala et al., 2026). The results are reported below (only for the exposed cohort of interest) and summarized in Table 4.

**Table 4:** Stress test summary (oncology - smoking cohort).

Parameter	Male light/moderate smokers (<16 cig/day)
Person-years (*PY*)	276,461
Observed events (*Y*)	206
Observed rate ( λ )	0.000745
*LL* (*Garwood*) - rate	0.000652
*SE* (*Poisson*)	0.0000519
Stress test Z-score	1.79
Indicative *p*	≅ 0.036

*Exposed Cohort* (Light/Moderate Smokers < 16 cig/day): Person-years *PY* = 276,461, observed events *Y* = 206, macro-incidence rate λ = 206/276,461 = 0.000745.

*Exact Garwood Lower Limit* (*Y* = 206): dflow = 412, $\chi^{2}(0.025,412)$ ≅ 360.48.

*LL*(*Garwood*)_rate_ = 180.24/276,461 = 0.000652.

*SE*(*Poisson*): $\sqrt{206}$ /276,461 ≅ 0.0000519.

Z-score: *Z* = ((0.000745–0.000652)/0.0000519) ≅ 1.79 (indicative one-tailed *p* ≅ 0.036).

Also for this third case, the resulting *Z*-score (1.79) remains below the 1.96 asymptotic boundary, illustrating that the structural margin of safety is thin.

In conclusion, across all three case studies, our structural stress test confirms that Wald’s asymptotic significance in massive biomedical databases may mask underlying inferential instability. By enforcing exact Poisson uncertainty floors, rather than Gaussian approximations, the framework provides a diagnostic filter to distinguish structurally valid signals from scale-induced outcomes.

## 4 Discussion

The empirical evaluations conducted across our three clinical case studies, spanning foundational autoimmune cohorts, multi-million-record PSM-balanced registry datasets, and large-scale oncological lifestyle investigations, reveal a kind of vulnerability in modern biomedical informatics and epidemiological research based on big data. When massive databases and advanced machine learning models are deployed to investigate rare outcomes, the resulting numerical outputs exhibit a sensitivity to sample size, revealing how asymptotic assumptions can influence inferential certainty.

A critical methodological distinction of our approach lies in the epistemological shift from descriptive interval-matching to an active stress test of resilience. Traditionally, exact methods like Garwood’s are employed purely to map non-symmetric confidence intervals around a point estimate. We moved further introducing a radical departure by utilizing the lower boundary of the exact Poisson distribution as a null benchmark: setting the null hypothesis to the lower limit calculated with the Garwood method.

We must explicitly acknowledge that Garwood did not propose this null hypothesis test; we do. Garwood’s original formulation was merely designed to calculate exact Poisson confidence limits. By repurposing Garwood’s methodology as a rigorous stress-test threshold, we establish a deliberate methodological intervention.

Why is this choice conceptually vital? Because it transforms the test from a passive geometrical check into an active survival verification under adversity. Instead of asking whether an interval contains a value, we ask: *Does the observed signal possess enough internal energy to survive against the absolute worst-case boundary of its own sampling distribution?*

In fact, as demonstrated by our comparative analyses, traditional survival models (such as Cox regressions combined with Wald tests) routinely churn out ultra-significant *p*-values (plunging well below 0.0001) often because huge sample sizes (*N* in the millions or massive person-years) act as a variance-reducer. They inflate the precision of the estimate by dividing the variance by a large denominator, shrinking the Wald standard error to near-zero.

Our inference stress test neutralizes this denominator issue. By substituting the asymptotic Wald standard error with the exact Poisson standard error, we anchor the dispersion strictly to the observed event count (*n* or *Y*). Consequently, when a nominally significant Hazard Ratio generated by a multi-million-record database is forced through our stress test, recomputed *Z*-values, often hovering near the 1.96 critical threshold, reveal that the initial signal lies close to the uncertainty floor.

It should be considered that obtaining stress-tested evaluations that do not clear the 1.96 asymptotic resistance threshold in cohorts exceeding millions of subjects is not a limitation of our method; it is rather an epistemological exposure of structural instability of the original dataset. If a biological signal were truly valid and physically consistent, it would maintain a secure structural buffer even when tested against the conservative floor of its own exact Poisson uncertainty. When a multi-million-record cohort yields a borderline or sub-threshold result under an exact Poisson load test, it proves that the original finding was a mathematical construct rather than possessing pure causal validity on its own. With this regard, it should be also reiterated that the formalization provided in Section 2.2 through our algebraic framework, elucidates perspective: proposing a load-test that bridges the gap between exact small-sample foundations and the realities of mega-cohort analytics.

Another crucial methodological nuance to be clarified regards the estimands. While multivariable adjustments (such as Propensity Score Matching and Cox proportional hazards models) estimate conditional Hazard Ratios rather than crude incidence rates, our test is not proposed as a competing causal estimator. Rather, it serves as an independent, model-agnostic structural verification. As suggested by the striking empirical convergence between the unadjusted initial Vitiligo cohort (case 1) and the 1:4 PSM-balanced extended dataset (case 2), this structural vulnerability persists regardless of modeling sophistication. This suggests that the observed limitation may be a fundamental reflection of event scarcity, not an artifact of model selection. In fact, regardless of analytical complexity, if the underlying count data fails to maintain adequate structural distance from its exact Poisson uncertainty floor, the perceived certainty of the big data output remains exposed to scale-induced fragility.

All this said, certain limitations must be noted.

First, our analysis focuses on only three prominent case studies (dermatology and oncology). However, we strongly suspect that this pathology is not isolated to these domains; rather, the entire recent biomedical literature is presumably subject to this asymptotic limitation wherever rare events meet massive digital cohorts.

Second, certain analytical Poisson assumptions underlying our test warrant brief mention. It is true that binary event counts in fixed cohorts are natively governed by a Binomial distribution and this could be seen as a limitation of our approach. Nonetheless, in the rare-event regime typical of large-scale cohorts, Binomial and Poisson frameworks practically converge, justifying our exact Poisson baseline. Moreover, the exact Garwood lower limit assumes a pure Poisson process (constant underlying event rate without unmeasured overdispersion or clustering). While this structural assumption makes our stress test highly conservative, it serves as a baseline safeguard against scale-induced false positives, leaving researchers mindful that extra-Poisson variability must always be evaluated in complex clinical data.

## 5 Conclusion

The current big data paradigm in clinical AI rests on a precarious foundation: the uncritical application of asymptotic statistics to rare phenomena. By demonstrating that ultra-significant Wald-based *p*-values can be amplified by sample size, this work proposes a necessary epistemological reset. Our proposed stress test, anchored in the exact Poisson distribution, and evaluated against the rigorous 1.96 asymptotic resistance threshold, serves as a critical diagnostic tool, prompting modern epidemiology to recognize that a signal, no matter how large the cohort, is often as valid as its resilience against the fundamental stochasticity of events.

In an era where algorithms are increasingly tasked with driving life-critical decisions, adopting this approach to data validation provides a valuable methodology to assess the structural resilience of clinical signals independently of database scale. It should be also recognized that other valid alternatives exist, such as those that emphasize issues of uncertainty quantification in big medical data across frequentist and Bayesian paradigms (Cappi et al., 2022; Fan et al., 2024), thereby confirming that a rigorous auditing of finite-sample performance remains an essential safeguard for reliable biomedical discovery, especially when the relative knowledge is used as a learning basis for medical AI.

## Data Availability

The original contributions presented in the study are included in the article/supplementary material, further inquiries can be directed to the corresponding author.

## References

[ref1] Agresti A. Coull B. A. (1998). Approximate is better than exact for interval estimation of binomial proportions. Am. Stat. 52, 119–126. doi: 10.1080/00031305.1998.10480550

[ref2] Alhumaidi N. H. Dermawan D. Kamaruzaman H. F. Alotaiq N. (2025). The use of machine learning for analyzing real-world data in disease prediction and management: systematic review. JMIR Med. Inform. 13:e68898. doi: 10.2196/68898, 40537090 PMC12226786

[ref3] Cappi R. Casini L. Tosi R. Roccetti M. (2022). Questioning the seasonality of SARS-COV-2: a Fourier spectral analysis. BMJ Open 12:e061602. doi: 10.1136/bmjopen-2022-061602, 35443965 PMC9021461

[ref4] Fan K. Subedi S. Yang G. Lu X. Ren J. Wu C. (2024). Is seeing believing? A practitioner’s perspective on high-dimensional statistical inference in Cancer genomics studies. Entropy 26:794. doi: 10.3390/e26090794, 39330127 PMC11430850

[ref5] Garwood F. (1936). Fiducial limits for the Poisson distribution. Biometrika 28, 437–442. doi: 10.1093/biomet/28.3-4.437

[ref6] Habehh H. Gohel S. (2021). Current genomics, machine learning in healthcare. Curr. Genomics 22, 291–300. doi: 10.2174/1389202922666210705124359, 35273459 PMC8822225

[ref7] Kim H. J. Kim M.-H. Chun E. M. (2026). Reply. J. Allergy Clin. Immunol. 158, 614–617. doi: 10.1016/j.jaci.2026.06.009 42556911

[ref8] Kim H. J. Kim M.-H. Park S. J. Choi M. G. Chun E. M. (2025). Autoimmune adverse event following COVID-19 vaccination in Seoul, South Korea. J. Allergy Clin. Immunol. 153, 171–1720. doi: 10.1016/j.jaci.2024.01.025 38520423

[ref9] Roccetti M. (2026a). Unlocking the stochastic parrot: epistemic obligation and the decline of biological plausibility in clinical reality. Inform. Med. Unlocked. 62:101752. doi: 10.1016/j.imu.2026.101752

[ref10] Roccetti M. (2026b). Logical paradoxes and epidemiologic inconsistencies in vaccine-related vitiligo risk assessment. J. Allergy Clin. Immunol. 158, 614–614. doi: 10.1016/j.jaci.2026.02.023 41842858

[ref11] Rothman K. J. Lash T. L. Greenland S. (2008). Modern Epidemiology. Philadelphia, PA: Lippincott Williams & Wilkins.

[ref12] Sala I. Botteri E. Berstad P. Fontvieille E. Zouiouich S. Sánchez M. J. . (2026). Association between lifestyle and risk of early-onset cancer: evidence from the European prospective investigation into cancer and nutrition and the UK biobank. Eur. J. Cancer 243:116869. doi: 10.1016/j.ejca.2026.116869 42269246

[ref13] Therneau TM (1997) Extending the cox model. In: Lin D.Y. Fleming T.R. (Eds.) Proceedings of the First Seattle Symposium in Biostatistics, Lecture Notes in Statistics. New York, NY: Springer

